# COVID-19 Communication From Seven Health Care Institutions in North Texas for English- and Spanish-Speaking Cancer Patients: Mixed Method Website Study

**DOI:** 10.2196/30492

**Published:** 2021-08-31

**Authors:** Robin T Higashi, John W Sweetenham, Aimee D Israel, Jasmin A Tiro

**Affiliations:** 1 University of Texas Southwestern Medical Center Dallas, TX United States; 2 Harold C Simmons Comprehensive Cancer Center Dallas, TX United States

**Keywords:** COVID-19, coronavirus, safety net, internet, communication

## Abstract

**Background:**

The COVID-19 pandemic has created an urgent need to rapidly disseminate health information, especially to those with cancer, because they face higher morbidity and mortality rates. At the same time, the pandemic’s disproportionate impact on Latinx populations underscores the need for information to reach Spanish speakers. However, the equity of COVID-19 information communicated through institutions’ online media to Spanish-speaking cancer patients is unknown.

**Objective:**

We conducted a multimodal, mixed method document review study to evaluate the equity of online information about COVID-19 and cancer available to English- and Spanish-speaking populations from seven health care institutions in North Texas, where one in five adults is Spanish-speaking. Our focus was less on the “digital divide,” which conveys disparities in access to computers and the internet based on the race/ethnicity, education, and income of at-risk populations; rather, our study asks the following question: to what extent is online content useful and culturally appropriate in meeting Spanish speakers’ information needs?

**Methods:**

We reviewed 50 websites (33 English and 17 Spanish) over a period of 1 week in the middle of May 2020. We sampled seven institutions’ main oncology and COVID web pages, and both internal (institutional) and external (noninstitutional) linked content. We conducted several analyses for each sampled page, including (1) thematic content analysis, (2) literacy level analysis using Readability Studio software, (3) coding using the Patient Education and Materials Assessment Tool (PEMAT), and (4) descriptive analysis of video and diversity content.

**Results:**

The themes most frequently addressed on English and Spanish websites differed. While “resources/FAQs” were frequently cited themes on both websites, English websites more frequently addressed “news/updates” and “cancer+COVID,” and Spanish websites addressed “protection” and “COVID data.” Spanish websites had on average a lower literacy level (11th grade) than English websites (13th grade), although still far above the recommended guideline of 6th to 8th grade. The PEMAT’s overall average accessibility score was the same for English (n=33 pages) and Spanish pages (n=17 pages) at 82%. Among the Dallas-Fort Worth organizations, the average accessibility of Spanish pages (n=7) was slightly lower than that of English pages (n=19) (77% vs 81%), due mostly to the discrepancy in English-only videos and visual aids. Of the 50 websites, 12 (24%) had embedded videos; however, 100% of videos were in English, including one on a Spanish website.

**Conclusions:**

We identified an uneven response among the seven health care institutions for providing equitable information to Spanish-speaking Dallas-Fort Worth residents concerned about COVID and cancer. Spanish speakers lack equal access in both diversity of content about COVID-19 and access to other websites, leaving an already vulnerable cancer patient population at greater risk. We recommend several specific actions to enhance content and navigability for Spanish speakers.

## Introduction

The COVID-19 pandemic created an urgent need for information to reach people with cancer because they are twice as likely to contract COVID-19 [1] and eight times more likely to die from it [2]. Health care institutions’ websites constitute a major communication mechanism with the public at large and often have subsections to specifically serve the needs of cancer patients. As health care consumers, patients rely on these websites to provide information about available health services, basic information about health problems, and access to additional resources [3]. For patients with cancer, websites may include information about multidisciplinary cancer care (ie, chemotherapy, surgery, and radiation), support services (eg, case management), and survivorship resources (eg, wellness education). During public health crises, timely and equivalent access to health information is critical for patients to adequately inform and protect themselves. However, the equity of information about COVID-19 to Spanish-speaking cancer patients, which is communicated through institutions’ online media, is unknown.

The disproportionate impact of the pandemic on healthy Latinx populations [4,5] underscores the need for equally high-quality information to reach Spanish-speaking populations impacted by cancer and concerned about COVID. The Centers for Disease Control and Prevention (CDC) estimates that the risks of COVID-19 infection, hospitalization, and death among Latinx persons were 2.0, 3.0, and 2.3 times higher compared to those among non-Hispanic Whites [6]. Much of this was likely due to the fact that Latinx populations are at elevated risk for severe disease given their higher rates of comorbid conditions [4] and exposure due to living and working conditions [5]. A study in May 2020 on access to coronavirus testing in major Texas cities also suggested that lack of testing locations in heavily Latinx and African American neighborhoods may have hampered quarantine efforts, enabling the virus to spread unchecked and contributing to disproportionate rates of COVID-19 [7].

Health care institutions serving large Spanish-speaking populations have a professional and moral obligation to ensure that information reaches Spanish speakers. Moreover, the disproportionate impact of the pandemic on Black and Latinx populations [4,5] in general elevates the need for information to reach Spanish-speaking populations impacted by cancer and concerned about COVID. For example, the Dallas-Fort Worth (DFW) area is home to over 1.6 million Spanish speakers [8], that is, persons aged 5 years or older who speak Spanish at home. Spanish speakers, the largest non-English speaking group, comprise approximately 21% of the 7.5 million residents in the 13-county DFW catchment area [9]. Among Spanish-speaking adults aged 25 years or older, most have less than a high school education (42%), 26% are high school graduates, and 14% are college graduates or beyond [10].

Educational attainment notwithstanding, the delivery of health information at a low literacy level (between the 6th and 8th grades) is a recommended best practice to enhance comprehension of materials [11]. While the average American adult reads at about an 8th grade level, the American Medical Association recommends that the readability of patient-facing health materials be no higher than 6th grade [12]. In the United States, adults who prefer to communicate in Spanish are especially affected by negative health outcomes associated with low literacy, such as higher emergency department utilization, higher morbidity, and lower use of preventive services [13-15]. Therefore, with new disease outbreaks like COVID, it is particularly important for new information to be conveyed in Spanish at a low literacy level. Thus, it is critically important to monitor if online information meets the needs of populations with lower literacy.

In this paper, our focus was less on the “digital divide,” which conveys disparities in access to computers and the internet based on the race/ethnicity, education, and income of at-risk populations [16-18]. We considered the following question: “Once Spanish-speaking consumers have physical access to technology, to what extent is the content of institutions’ websites useful and culturally appropriate in meeting their needs?” [19]. By “culturally appropriate,” we mean how language and messages are targeted to address the needs of the population. In this study, we examined several forms of cultural appropriateness, including availability of websites in Spanish, reading level, ease of locating information, and visual representations of racial/ethnic minorities [20,21].

We conducted a document review study to evaluate the equity of information about cancer and COVID-19 available online to English and Spanish speakers from large health care institutions in the DFW area. Document analysis is the ideal method to capture information at discrete periods of time as a historical record of the online information presented to health care consumers by each institution. It allows for thematic analysis using pattern recognition and fitness to the proposed purpose of the document. Here, we report the results of that evaluation, including a thematic analysis of institutional website content, measurement of literacy and accessibility, and analysis of links to external websites and representations of diversity.

## Methods

### Website Sampling

Rigorous document analysis involves a systematic sampling strategy grounded in the research problem and the purpose of the study [22]. In this exploratory study, our website sampling approach was guided by the goal of comparing what cancer- and COVID-related resources were available to English- and Spanish-speaking consumers in the DFW area. We sampled a total of 50 websites in a hierarchical sampling “block” strategy with the criteria outlined in Table 1.

**Table 1:** Website sampling by blocks, criteria, and number of pages.

Sampling block	Pages	Criteria	Number of pages (N=50)
A	Seven DFW^a^ institutions’ main cancer/oncology websites	We sampled all seven institutions’ main cancer/oncology pages in English. Only one institution had a parallel^b^ Spanish website, which we also sampled (ie, we did not sample Google translate versions of English websites).	8
B	Seven DFW institutions’ main COVID websites	Same criteria as Block A (n=8). In addition, one academic medical center had two COVID websites to orient patients/families on health care services and inform the public about research and educational missions, so we sampled both in English.	9
C	Internal direct sublinks to English/Spanish parallel content	We sampled any internal^c^ linked content in Spanish from the main cancer or COVID pages if (1) parallel English and Spanish contents were available for comparison; and (2) relevant^d^ information was available.	10 (5 pairs)
D	External direct sublinks to English/Spanish parallel content	We sampled any external^e^ linked parallel content in Spanish and English from the main cancer or COVID pages using the same criteria as in Block C.	12 (6 pairs)
E	External direct sublinks of English or Spanish nonparallel content	We sampled external nonparallel English (n=7) and Spanish (n=4) pages with relevant information linked from the main cancer or COVID pages.	11

^a^DFW: Dallas-Fort Worth.

^b^“Parallel” is defined as separate web pages that mirror each other in format and content.

^c^“Internal links” are links to other web pages authored by the institution.

^d^“Relevant” is defined as including information about COVID that would be pertinent specifically to a cancer patient, survivor, or someone participating in a cancer prevention service.

^e^“External links” are links to web pages not authored by the institution.

We focused first on the main oncology and COVID web pages of a purposive sample of seven prominent DFW area health care institutions (Blocks A and B) to assess the type and accessibility of COVID and cancer information available to consumers. As shown in Table 2, our purposive sample included the two safety-net institutions in the metropolitan area (Institutions 1 and 2), a nonprofit cancer specialty health provider network (Institution 3), and the four largest nonprofit health systems (Institutions 4-7a), including one academic health system, with “largest” defined by the number of hospital beds. These seven organizations represent all but one of the top 10 cancer care provider organizations in DFW (excluded one, a private health system) [23].

**Table 2:** Description of the seven institutions sampled.

Institution number	Description
1	Safety net
2	Safety net
3	Nonprofit cancer specialty health provider network
4	Nonprofit health system
5	Nonprofit health system
6	Nonprofit health system
7/7a	Nonprofit academic health system/Affiliated academic comprehensive cancer center

From there, we sampled these seven institutions’ internal and external linked contents available in both languages to further assess the equity of information to English- and Spanish-speaking consumers (Blocks C, D, and E). By “internal links,” we mean links to other web pages authored by the institution. As indicated in the sampling strategy, we assessed internal linked content if (1) parallel English and Spanish content was available for comparison and (2) content was relevant. By “parallel,” we mean separate web pages in English and Spanish designed by the institution to convey the same information. We define “relevant” as including information about COVID that would be pertinent specifically to a cancer patient, survivor, or someone participating in a cancer prevention service. Using this strategy, we identified five pairs (n=10 websites) of internal English and Spanish content from two institutions’ main COVID websites, and six pairs (n=12 pages) of parallel English and Spanish external linked websites. These are itemized as Blocks C and D in Table 1. Finally, we sampled external links to seven English and four Spanish nonpaired websites to assess potentially inequitable information available to consumers (Block E). The 50 total websites included 33 (66%) predominantly English and 17 (34%) Spanish websites.

### Data Collection

To promote systematic and consistent data collection, the principal investigator (RTH) designed a data collection tool in REDCap [24] that structured rules for evaluation and data entry of specific constructs informed by the literature [12,19,25-27]. The tool consisted of (1) topics for thematic analysis (eg, main headers, presence of embedded videos, internal and external links, and markers of cultural inclusiveness); (2) a literacy score, measured using *Readability Studio* (Oleander Solutions) software; and (3) a survey instrument consisting of 12 items measuring accessibility (using the Patient Education and Materials Assessment Tool [PEMAT] [28]). We also assessed websites with respect to markers of diversity and inclusivity in video and visual content. One research staff used the tool to collect data from each website, except for the PEMAT survey portion, which was completed by two staff members per website to enhance rigor.

All website data were collected during a 1-week period in the middle of May 2020. At that time, all 50 websites had been updated in 2020. Of the 50 websites, 31 were updated since March 1, 2020; 26 were updated since April 1, 2020; and 12 were updated since May 1, 2020. A few websites reported being updated daily.

The principal investigator performed a quality assessment check by reviewing 10% of the collected data to ensure completeness and adherence to the data collection tool during documentation.

### Data Analyses

Once data were collected, several analyses were performed, including: thematic content analysis, literacy level analysis using Readability Studio software, coding of the PEMAT, and descriptive analysis of video and diversity content.

#### Thematic Content Analysis

We used website headers to approximate the thematic content of the main COVID and cancer websites in English and Spanish. First, data collection staff recorded the headers that corresponded to content related to cancer or COVID on each of the 50 selected websites. Next, the principal investigator reviewed these data and created a qualitative codebook consisting of 29 topics, such as “prevention,” “resources,” and “testing.” Three persons then double coded in an alternating matrix the free-text headers into codebook topics. Discrepancies between two coders were resolved by the principal investigator. A table of this conversion process is shown in Multimedia Appendix 1. We used the same approach to analyze the thematic content of linked internal and external pages from the institutions’ main COVID and cancer websites to assess health care consumers’ ease of navigating to additional information.

#### Literacy Level

A literacy level or readability score approximates the level of education a person may need to be able to read a piece of text easily. Scores are generally based on factors such as sentence length, syllable length, and syntax. Website content was scored using *Readability Studio,* which yielded a combined score from the Gilliam-Peña-Mountain and SOL (Spanish SMOG) readability scales [29,30]. For the purpose of this study, we defined “low literacy” as a reading level less than 9th grade.

#### PEMAT

The PEMAT instrument measures the overall clarity and accessibility of print materials, such as the simplicity of concepts, syntax, layout, and the availability of nontext communication tools [28]. Four coders were trained by the lead investigator in the use of the PEMAT instrument to promote consistency in coding. Then, two pairs of two coders each scored 25 websites (for a total of 50 websites). Coding agreement between paired individuals was high (*k*=0.77 and 0.82). Where discrepancies existed, coders reconvened, discussed, and decided upon one code to be used for the final PEMAT scoring.

#### Video Content

While most individuals learn visually (ie, what they see and read), others are auditory or kinesthetic learners, which means they prefer to learn by touch or manipulation like note-taking and role-playing. Given these different learning styles, videos can enhance the accessibility of websites by engaging audiences, reducing literacy burden, and quickly delivering important health messages [31]. We counted the number of websites that contained embedded videos and language videos, in which videos were presented.

#### Diversity and Inclusiveness

Communications of racial and ethnic diversity on websites can convey an institution’s core values and may serve to attract members of racial/ethnic minorities to web content [27,32]. Therefore, we counted the number of websites with pictures of persons of perceived non-White racial backgrounds, or statements of diversity (eg, “For interpreting services, please call: xxx-xxxx.”) as markers of cultural inclusiveness.

## Results

### Spanish Content Availability

Only Institution 1 from the seven institutions had parallel Spanish cancer and COVID websites. In 2015, the institution logged 75,000 encounters each month with non-English speakers, 92% of which were with Spanish-speakers [33]. Institutions 3, 4, and 7a had some Spanish content and/or links to external websites in Spanish. The remaining websites had no Spanish content and no links to external Spanish content.

### Thematic Content

**Figure 1:**
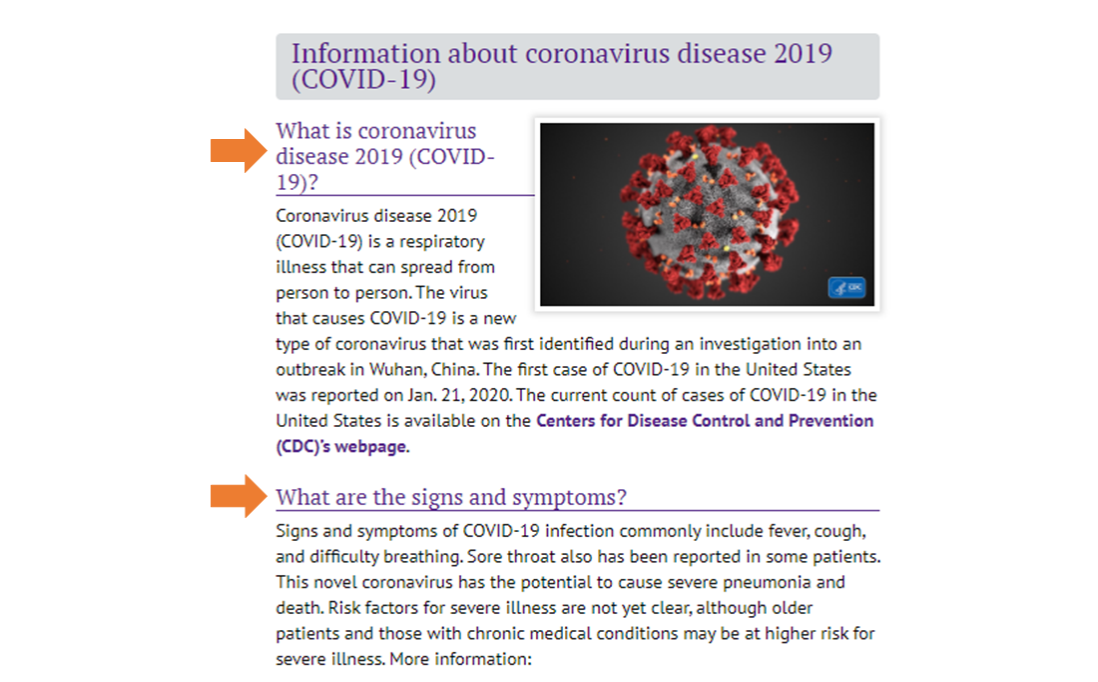
Example of an English website header.

**Figure 2:**
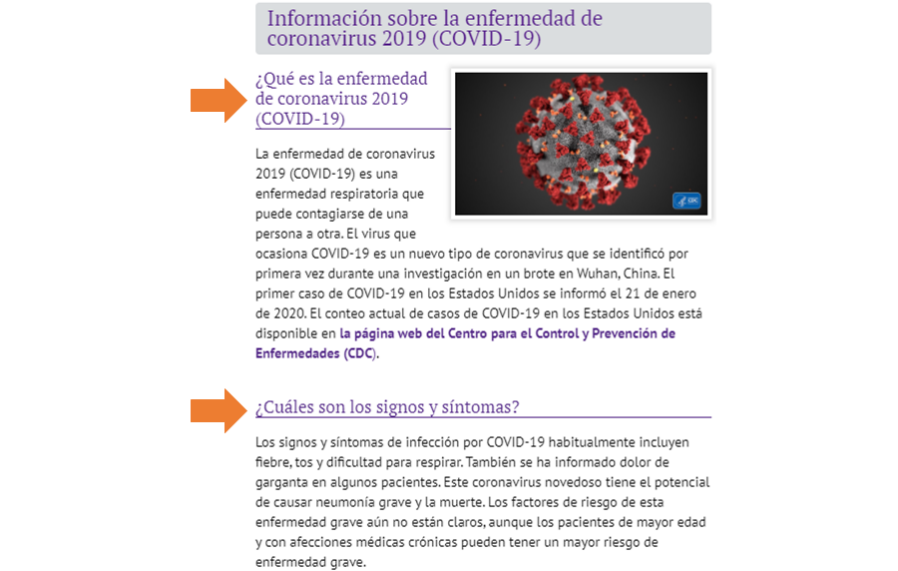
Example of a Spanish website heading.

The top 10 themes of website headers are reported by language and number of times cited in Table 3. Examples of website headers are shown in Figures 1 and 2.

**Table 3:** Themes of website headers.

Website language and theme		Value, n (%)
**English websites (n=33)**		
	Resources/more information/FAQ	25 (76)
	Updates/news^a^	20 (61)
	Cancer and COVID	19 (58)
	Prevention/how it spreads	14 (42)
	Protection/what you can do	13 (39)
	Services/treatments available	13 (39)
	Signs/symptoms^a^	13 (39)
	Testing/screening^a^	11 (33)
	What is X institution doing?^a^	10 (30)
	What to do if you are sick or you think you have COVID	10 (30)
**Spanish websites (n=17)**		
	Protection/what you can do	14 (82)
	Resources/more information/FAQ	8 (47)
	COVID data^b^	7 (41)
	What to do if you are sick or you think you have COVID	7 (41)
	Cancer and COVID	6 (35)
	Hours/locations/info for patients and visitors^b^	5 (29)
	Prevention/how it spreads	5 (29)
	Risk factors/risk assessment/high risk populations^b^	5 (29)
	Social distancing^b^	5 (29)
	Specific population information^b^	5 (29)

^a^Themes in English that did not appear in the top 10 themes cited on Spanish websites.

^b^Themes in Spanish that did not appear in the top 10 themes cited on English websites.

There were some similarities and several differences in the content of website information on English and Spanish websites. With respect to organizations’ main COVID websites, for example, content related to the topic of how to obtain additional resources (eg, where to find more information, and responses to frequently asked questions) figured prominently on both English (#1) and Spanish (#2) websites, as did content related to COVID prevention and protection. However, while COVID news and updates constituted the second most frequently cited theme and testing constituted the eighth most frequently cited theme in English, neither theme ranked among the top 10 content areas in Spanish. In contrast, the Spanish websites contained several headers and subheaders that did not figure in the top 10 English content areas, including COVID data, hours and locations for patients and visitors, risk factors or information for high-risk populations, social distancing, and information for specific populations (eg, elderly persons or pregnant women).

With respect to information specifically about COVID and cancer, there was far more information available in English (9/16, 56%) than in Spanish (4/16, 25%) among the institutions’ internal websites. Institutions 1, 2, and 3 had no information about COVID on their main cancer page; Institutions 4, 5, and 6 included a banner at the top of the page with a link to some form of COVID-19 information, such as “COVID-19 updates” and “Important information about COVID-19;” and Institution 7 had 21 instances of “COVID” mentioned on its cancer main page (represented as Institution 7a), indicating a substantial amount of detailed information for cancer patients. Examples of content headers included “Cancer and COVID-19: What You Need to Know,” “Virtual Visits Available,” and “Am I considered immunocompromised if I have had cancer treatment?” These headers linked consumers to content that, for example, explained safety measures for in-person care and informed patients of options for virtual care.

### Internal Links

**Figure 3:**
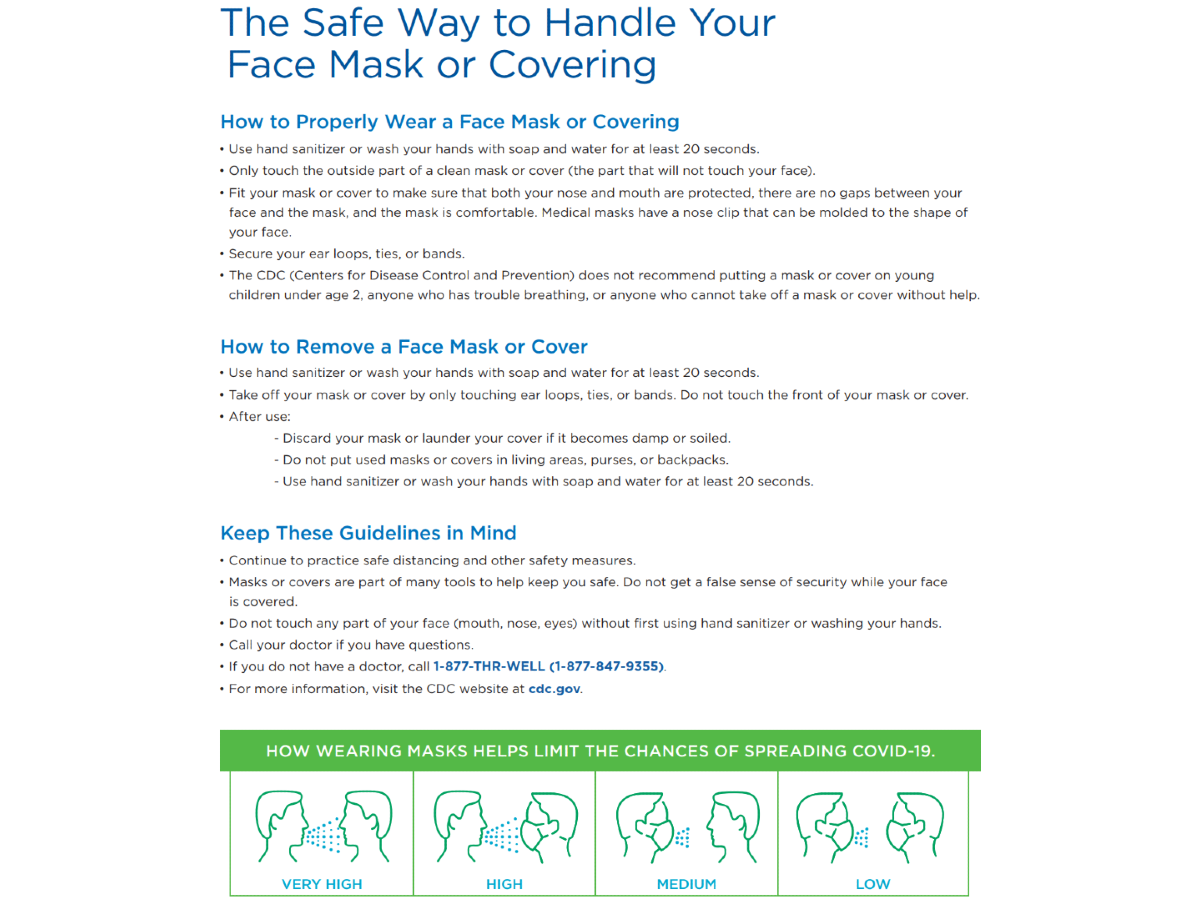
Example of an internally linked bulletin.

Institutions 3 and 4 had some internal content in English and Spanish, for example, a bulletin called “About Coronavirus” (Figure 3). A total of five pairs of English/Spanish links were identified (sampling Block C). Institutions 2, 5, 6, and 7 had no internal links or links to external Spanish content. However, for those familiar with how to access the function in the Chrome web browser, a “Google Translate” version of the website was available. An evaluation of the “Google Translate” versions of websites was beyond the scope of this study [34].

### External Links

For Institutions 1, 3, and 4, the main COVID website also contained links to external websites in Spanish, either as English/Spanish pairs (sampling Block D) or individual Spanish links (sampling Block E).

**Figure 4:**
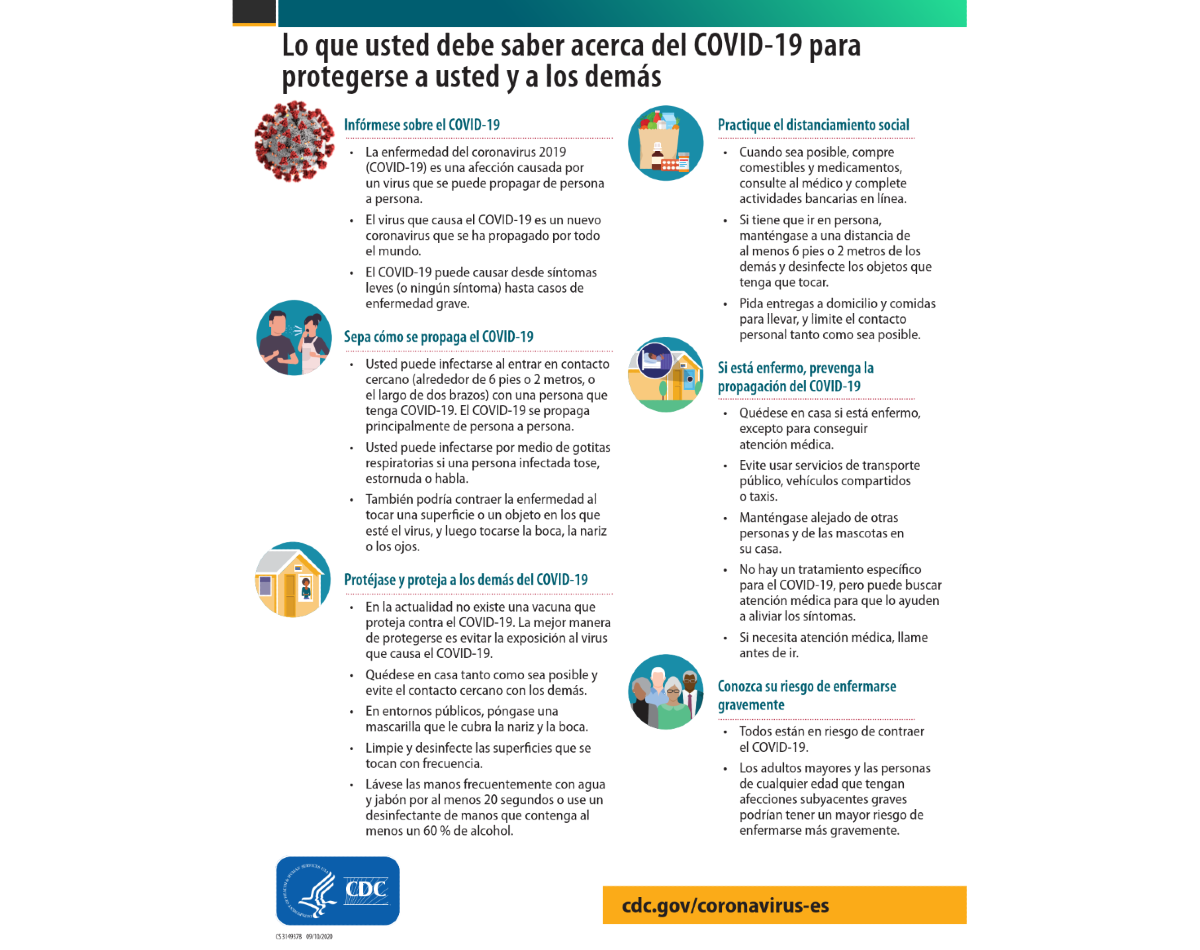
Example of a static factsheet.

Among all external links in Spanish (n=10), the main CDC coronavirus website was linked to twice, and 7 of 10 links were to CDC pages. However, 4 of 10 external links were to one-page static factsheets and not websites (Figure 4). Moreover, 4 of 5 links were to an English page (eg, [35]), where the user must locate a button to convert the page to Spanish, rather than linking directly to the Spanish website URL (eg, [36]). In other words, much of the available content in Spanish was abbreviated and did not lead to further opportunities to link to other websites, and of the available websites, many required users to navigate in English to arrive at Spanish text. Consumers with lower health literacy or digital literacy [37] may miss the opportunity to arrive at the Spanish website, or at best, may get frustrated in navigating there.

The external links from the cancer and COVID websites in English (n=24) from all seven DFW organizations demonstrated greater heterogeneity. In total, links included 10 different CDC websites, as well as local organizations (eg, Department of State Health Services, Komen Greater Fort Worth), academic organizations (eg, New England Journal of Medicine and American Society of Microbiology), and government entities (eg, National Institutes of Health, Occupational Safety and Health Administration). As was the case in Spanish, the main CDC coronavirus website was the most frequently linked-to page (four times); this was followed closely by the CDC’s website about symptoms (three times). All organizations linked to at least one CDC page. However, the fact that different, that is, nonparallel, websites were offered as links on the English and Spanish main pages underscores the finding that inequitable information is available to English- and Spanish-speaking consumers.

### Literacy Level

Among the 50 websites, the average literacy score for the English websites (n=33) was 13.2, while the average score for the Spanish websites (n=17) was lower at 11.7. Websites with parallel English and Spanish content scored exactly the same, at an overall average of 12.8. However, among the DFW area organizations’ main cancer and COVID websites only, the average literacy score was higher at 15.4 (range 14.8-16.9) and 12.6 (range 8.3-15.5), respectively. Only Institution 1’s main COVID page in Spanish had a low literacy level (score 8.3).

### PEMAT

The overall average accessibility score using the PEMAT analysis was the same for English (n=33 pages) and Spanish pages (n=17 pages) at 82%. Among the DFW organizations, the average accessibility of the Spanish pages (n=7) was slightly lower than that of the English pages (n=19) (77% vs 81%), due mostly to the discrepancy in English-only videos and visual aids. Overall, the most common items on which websites scored negatively included the following (the first and third items likely accounted for the high overall literacy scores):

- “Medical terms are used only to familiarize the audience with terms. When used, medical terms are defined” (22/50 websites scored negatively). Nondefined higher literacy terms included “oncology,” “SARS CoV-2,” and “intravenous iron supplementation.”
- “The material uses visual aids whenever they could make content more easily understood” (17/50 websites scored negatively). Visual aids included, for example, the use of videos, icons, graphics, and GIFs.
- “The material uses common everyday language” (9/50 websites scored negatively). Higher literacy language typically included multiple sentences containing more than 23 words and multiple words with more than three syllables, e.g. infrastructure.

In contrast, among the external organizations’ websites, the average accessibility score of the Spanish pages (n=10) was slightly higher than that of the English pages (n=13) (86% vs 83%). Overall, the scores indicate a moderately high level of accessibility. With relatively few total pages being scored, we could not state whether these differences were statistically significant.

### Video Content

Of the 50 websites, 12 (24%) had embedded videos in them; however, 100% of the videos were in English, including one that was on a Spanish website. This indicates a missed opportunity to not only reach Spanish speakers, but also engage lower literacy audiences using nontextual information delivery.

### Diversity and Inclusiveness

Just over half (n=26, 52%) of the 50 websites had pictures of people. Of those that had pictures, 69% (n=18/26) included people of non-White racial or ethnic backgrounds. Other markers of inclusivity included data stratified by race and ethnicity, patient stories told from the perspective of those from different racial backgrounds, and documents offered in several languages in a drop-down menu.

Missed opportunities were as follows: a button for a Spanish website was listed at the very bottom of an English web page, and the Spanish website about local cases was not updated in real time like the English equivalent (the Spanish website reported zero cases in the county as of March 2020, whereas the English website correctly reported the cases).

Lastly, there were a few instances where websites lacked equity or cultural sensitivity. By “cultural sensitivity,” we mean cultural awareness and appreciation for the needs of Spanish-speaking persons. These included the following:

- A link from to a YouTube video from the Spanish text “Pasos simples para prevenir COVID” (English translation: Simple steps to prevent COVID) took the user to a video in English, even though the text and page was in Spanish.
- Charts available on the English website (“Cases by Race and Age” and “Cases by Ethnicity and Age”) were not available on the Spanish parallel website.
- The English website was last updated the day before, whereas the Spanish parallel website was updated over a month ago. This resulted in significantly outdated and imbalanced content on the Spanish website.
- The English website used the phrase “Keep America Open,” whereas the Spanish parallel website said “Keep the United States open.” In this case, the English website lacks sensitivity because using the term “America” to refer to the United States implies a political and cultural dominance over a continental area.

## Discussion

This document analysis of seven health care institutions’ websites demonstrates that Spanish speakers lack equal access to information about COVID-19 compared with their English-speaking counterparts, leaving an already vulnerable cancer patient population at greater risk. In addition to a greater volume of information, English speakers had access to a wider variety of content via linked information on dynamic web pages rather than static fact sheets. Additionally, video content, which is recommended for low literacy audiences, was available only in English or on English websites. This is especially concerning given the finding of a recent study using nationally representative data that Hispanics were more likely to report watching health-related videos [38]. Moreover, findings noted postaccess disparities [19], such as ease of navigability, which could exacerbate deficits in content for Spanish-speaking consumers of online information. A summary of our main findings demonstrating the inequity of online information about cancer and COVID-19 available to English and Spanish speakers is shown in Table 4.

Our readability analysis demonstrated that overall Spanish websites had a lower average literacy level than English websites (11.7 vs 13.2). However, both literacy levels are unacceptably high given the recommended 6th to 8th grade reading level range for patient-facing health materials [12]. This indicates a significant need for institutional changes to make all websites more accessible to health care consumers in accordance with suggested guidelines [39].

According to the American Hospital Association’s Code of Ethics, health care institutions have professional and moral obligations to provide communications that are “clear, accurate, and sufficiently complete,” and “should be aimed primarily at better public understanding of health issues, the services available to prevent and treat illness, and patient rights and responsibilities to health care decisions” [40]. However, the guidelines are unclear regarding how health care institutions should best structure and deliver content during public health emergencies, such as the COVID-19 pandemic, when information is rapidly evolving and institutions may lack the resources for regular updates. The American Hospital Association guidelines also lack specificity about the scope and speed with which to inform the non-English-speaking public, which is critical in the DFW metroplex, where 21% of the population is Spanish-speaking. Indeed, findings from this study demonstrate an uneven response among the seven health care institutions to providing equitable information to Spanish-speaking DFW residents concerned about COVID and cancer. Variations in the proportion of Spanish-speaking patients served, institutional resources, organizational culture, and other factors may all play roles in these differences. Above all, however, there is a clear need for public health communication to reach vulnerable populations in real time. Our findings are consistent with the findings of a recent study by the National Cancer Institute (NCI) that found, during the early months of the COVID-19 pandemic, cancer survivors and caregivers were more likely to engage with NCI’s Cancer Information Services resources than tobacco users or the general public. This pattern was consistent for English- and Spanish-speaking users accessing content via telephone, instant messaging, email, and social media [41].

This study is unique in assessing the equity of local health care institutions’ cancer and COVID website content for English- and Spanish-speaking consumers. We are aware of only one other study that completed a limited examination of equity by noting accessibility of NCI comprehensive cancer centers’ visitor policies; it determined that the majority (66%) of the cancer centers published their visitor policies only in English, even in areas of the country with large proportions of Hispanic/Latinx populations [42]. Other studies have inventoried online resources about COVID-19 in Spanish, but these were limited in investigating the educational activities of health care institutions, namely instructional videos [43,44].

**Table 4:** Summary of the findings.

Variable			Finding for English speakers		Finding for Spanish speakers
**Health care institutions’ website availability**					
	Main cancer website	7 institutions		1 institution	
	Main COVID website	7 institutions		1 institution	
	Additional information via internally linked pages	7 institutions		3 institutions	
	Additional information via externally linked pages	7 institutions		4 institutions	
**Thematic content frequency**					
	#1 cited theme	Resources/more information/FAQ (76% of websites)		Protection/what you can do (82% of websites)	
	#2 cited theme	Updates/news (61% of websites)		Resources/more information/FAQ (47% of websites)	
	#3 cited theme	Information about cancer and COVID (58% of websites)		COVID data (41% of websites)	
**Usability**					
	Ease of navigability	Direct links		Page links to English requiring the user to locate the Spanish version via a pull-down menu or page links to Spanish at the bottom of the page	
	Diversity of information	Links to 24 different pages		Links to six different pages	
	Completeness of information	All 24 links are to “live” pages with additional links to further information		Four of six links are to limited static pages (eg, a PDF)	
**Literacy level^a^ and accessibility^b^**					
	Average literacy score overall	13.2 (n=33)		11.7 (n=17)	
	Average literacy score for institutions’ main cancer and COVID pages	15.4 (n=17)		12.6 (n=2)	
	Average accessibility score overall (DFW^c^ and external organization pages)	82% (n=33)		82% (n=17)	
	Average accessibility score among DFW institutions’ pages	81% (n=19)		77% (n=7)	
	Average accessibility score among external organization pages	83% (n=13)		86% (n=10)	
**Video content**					
	Availability	12/50 (24%) websites		0/50 (0%) websites	
**Diversity and inclusion (images of people^d^)**					
	Perceived non-White and White racial/ethnic groups	10/33 (30%) websites		8/17 (47%) websites	
	Only perceived White racial/ethnic groups	7/33 (21%) websites		1/17 (6%) websites	

^a^Literacy level measured by Readability software (Oleander Solutions).

^b^Accessibility measured by the Patient Education and Materials Assessment Tool (PEMAT).

^c^DFW: Dallas-Fort Worth.

^d^Remaining websites had no images of individuals (49% of 33 English websites; 47% of Spanish websites).

**Figure 5:**
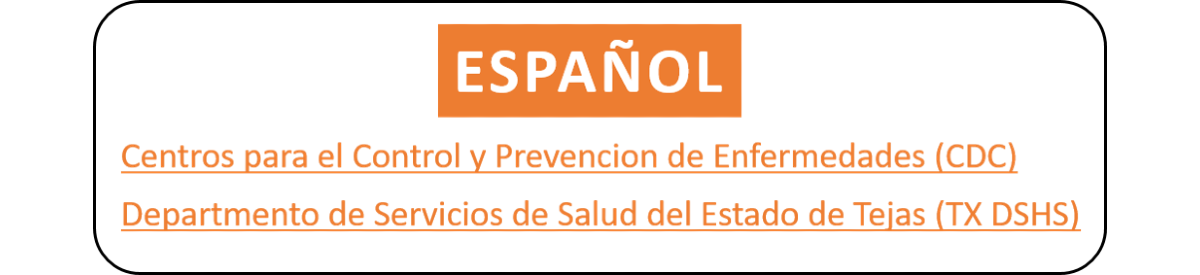
Example of linked content labeled in Spanish.

We recommend several specific actions to enhance content and navigability for Spanish speakers. First, all health care institutions should feature at least the CDC’s and their State Public Health Department’s main coronavirus websites. For example, in this study, the CDC’s main COVID websites were the English website [35] and Spanish website [36]; and the Texas Department of State Health Service’s main coronavirus websites were the English website [45] and Spanish website [46]. In addition to having websites in English and Spanish, both organizations’ main COVID websites link to many other COVID-related websites in English and Spanish. Second, they should label links to Spanish websites with text in Spanish, as shown in Figure 5. Third, we recommend lowering the readability of website text to the recommended 6th to 8th grade reading level. Fourth, linked content should be sent directly to the Spanish version of a page (eg, [36]), rather than to the English version (eg, [35]), where users would need to navigate in English to a button or pull-down menu to select the Spanish page. Lastly, English websites should display more markers of cultural inclusivity, such as images of people of non-White racial/ethnic backgrounds.

This study has several limitations. First, this study was conducted in Texas, a border state with a large Spanish-speaking population. The findings may not be as generalizable or relevant for other regions with small Spanish-speaking populations. Second, this analysis was performed during a single week in May 2020; we did not examine how information evolved. For example, by May 2020, the country had experienced only the first wave of the pandemic; a cursory review in April 2021 revealed a greater volume of Spanish content on some health care institutions’ websites compared to when they were analyzed the year prior. Finally, this study, like all document review studies, is inherently limited in detail; intentionality cannot be clarified, as would be expected in an interview, for example [22]. For this reason, this analysis did not include an evaluation of translation accuracy and conceptual equivalence. However, document analysis studies like this one provide clear objective documentation of an institution’s online record that can be reanalyzed by others. Given that a website’s communication goal should be to meet the specific needs of local communities [47], further research using other qualitative methods could clarify whether there was an evidence-based rationale for differentiating content between English and Spanish websites.

The COVID-19 pandemic has presented significant challenges for health care institutions in meeting the informational needs of the public. This study is significant in being the first of its kind to demonstrate inequities in the online information available to English- and Spanish-speaking residents concerned about COVID and cancer in a large US metropolitan area. Future research should qualitatively assess where Spanish speakers go for information about COVID and cancer, and evaluate the implications of information-seeking from potentially nonreputable sources.

## Appendix

Multimedia Appendix 1 Coding of website headings.

## Abbreviations

CDC: Centers for Disease Control and Prevention
DFW: Dallas-Fort Worth
NCI: National Cancer Institute
PEMAT: Patient Education and Materials Assessment Tool
